# Serotonin transporter dependent modulation of food-seeking behavior

**DOI:** 10.1371/journal.pone.0227554

**Published:** 2020-01-24

**Authors:** Jianzheng He, Franziska Hommen, Nina Lauer, Sophia Balmert, Henrike Scholz

**Affiliations:** Albertus-Magnus University of Cologne, Department of Biology, Institute for Zoology, Cologne, Germany; Biomedical Sciences Research Center Alexander Fleming, GREECE

## Abstract

The olfactory pathway integrates the odor information required to generate correct behavioral responses. To address how changes of serotonin signaling in two contralaterally projecting, serotonin-immunoreactive deutocerebral neurons impacts key odorant attraction in *Drosophila melanogaster*, we selectively alter serotonin signaling using the serotonin transporter with mutated serotonin binding sites in these neurons and analyzed the consequence on odorant-guided food seeking. The expression of the mutated serotonin transporter selectively changed the odorant attraction in an odorant-specific manner. The shift in attraction was not influenced by more up-stream serotonergic mechanisms mediating behavioral inhibition. The expression of the mutated serotonin transporter in CSD neurons did not influence other behaviors associated with food seeking such as olfactory learning and memory or food consumption. We provide evidence that the change in the attraction by serotonin transporter function might be achieved by increased serotonin signaling and by different serotonin receptors. The 5-HT1B receptor positively regulated the attraction to low and negatively regulated the attraction to high concentrations of acetic acid. In contrast, 5-HT1A and 5-HT2A receptors negatively regulated the attraction in projection neurons to high acetic acid concentrations. These results provide insights into how serotonin signaling in two serotonergic neurons selectively regulates the behavioral response to key odorants during food seeking.

## Introduction

Animals such as mammals and insects heavily rely on the olfactory system to search for and detect a suitable food source. The olfactory system is a powerful system for decoding odor information that is relevant for the animal’s energy demands. The relevance of odor information to the animal might be assessed by examining the behavioral response to the odor information in a choice situation. Animals might respond with approach, avoidance or indifference.

A food source such as an apple emits a complex odor bouquet [1]. In addition to the fruit smell, yeast might settle on the surface, contributing its own typical smell of ethyl acetate (EtOAc) [2]. Furthermore, yeast converts fruit sugar into the odorant ethanol (EtOH). Ethanol in turn is converted into acetic acid (AA) by the bacteria acetobacter spec. [3]. The fruit fly *Drosophila melanogaster* is attracted to key odorants such as AA, EtOH and EtOAc within food odor blends [4], [5], [6]. The key odorants are first detected on the level of the olfactory receptor neurons (ORNs). AA strongly activates IR75a receptor-expressing ORNs [7], and EtOAc activates Or42b receptor-expressing ORNs [8]. At the ORN level, EtOH is recognized within food odor blends in an olfactory co-receptor (Orco)-dependent manner [5], [4]. The circuit-level mechanism by which the attraction to a key odorant within food odor blends is regulated is not understood.

Serotonin is a neurotransmitter and neuromodulator and influences olfactory information processing in mammals and insects [9], [10], [11]. In *Drosophila*, a single pair of contralaterally projecting serotonin-immunoreactive deutocerebral (CSD) neurons directly releases serotonin in the antennal lobes (AL) [12], [13]. The primary neurite of the CSD neurons projects through the ipsilateral antennal lobe to the mushroom body calyx (MB) and the lateral horn (LH) on both sides of the brain and terminates in the contralateral AL [13], [12]. The ALs are the primary relay station of the olfactory system consisting of a unique set of ORNs, local interneurons (LNs) and projection neurons (PNs) that transfer olfactory information to higher brain centers such as the LH and MB [14], [15], [16].

Loss of neuronal activity of the CSD neurons increases CO_2_ avoidance and the attraction to female pheromones for male flies [17]. Increased serotonin levels in adult flies act as negative regulator for olfactory attraction to low concentration of the key odorant ethanol within food odor mixtures [18], [19]. In addition, altered serotonin signaling in CSD neurons by expressing a serotonin transporter with mutated serotonin binding sites increases the attractiveness to a high ethanol concentration, which is normally neither attractive nor aversive, within food odor blends [18]. On the cellular level, exogenous serotonin enhances calcium signaling in projection neurons in an odorant-specific manner and enhances calcium signaling in inhibitory local interneurons [20]. Serotonin exerts its activity by binding to specific membrane-bound serotonin receptors. In *Drosophila*, five serotonin receptors including the 5-HT1A, 1B, 2A, 2B and 7 have been identified [21]. These receptors are differentially expressed in different subtypes of neurons within the ALs [22]. The action of serotonin within the synaptic cleft is terminated by reuptake by the serotonin transporter expressed on presynaptic serotonergic CSD neurons [4], [18]. Expression of a serotonin transporter with mutated serotonin binding sites results in reduced presynaptic serotonin concentrations in the soma of neurons. The expression of the mutated serotonin transporter in a subset of serotonergic neurons resulted in reduced attraction to ethanol enriched food odors, a behavior that is mimicked by opto-genetic activation of the same set of serotonergic neurons supporting that the expression of the mutated serotonin transporter results increased serotonin signaling [18].

Here, we address whether altered serotonin signaling in CSD neurons by expressing the mutated version of the serotonin transporter influences the response to key odorants within food odor blends. We address whether increased serotonin signaling rather than the reduction or loss of serotonin signaling mediates these behavioral changes. To determine the relevance of odor information for the behavior output of the animal, we used a binary choice assay consisting of two odor traps [6]. To understand how the serotonergic CSD neurons might regulate opposing behavioral responses on a circuit level, we performed neuroanatomical analysis. Since the CSD neurons synapse onto the MBs, a structure involved in the regulation of olfactory learning and memory, we next addressed whether short-term olfactory appetitive, aversive or aversive reversal learning and memory is altered by altered serotonin signaling in the CSD neurons. In addition, we asked whether altered odorant attraction influences food consumption. To investigate how the CSD neurons mediate differences in attraction, we analyzed the AA attraction of four different serotonin receptor mutants and flies with altered serotonin receptor function in GABAergic neuron or PNs. We provide evidence that 5-HT1B, 5-HT1A and 5-HT2A receptors are required to shape the response to key odorants such as AA in different sets of neurons.

## Material and methods

### Fly stocks

Flies were raised on ethanol-free standard cornmeal-molasses-yeast-agar medium at 25°C and 60% relative humidity with a 12-h / 12-h light—dark cycle. The following lines were used: *UAS-*BRP-short^straw^ [23]; *mb247-*DsRed*; mb247-*CD4::spGFP11, *UAS-*CD4::spGFP1-10 [24]; *LexAop-*CD4::spGFP11*; UAS-*CD4::spGFP1-10 [25]; *RN2-E-*Gal4 [26]; *Sert3-*Gal4 [18]; GAD1-Gal4 [27]; *UAS*-Sert^DN^::GFP [18]; *UAS-Trh-RNAi* [28]; *UAS*-mCD8::GFP [29]; *LexAop-*myr::mCherry [30]; *Orco*-LexA::VP16 [31]; and *GH146*-LexA::GAD [32]. The following stocks were provided by the Bloomington Stock Center: *GMR29A12-*LexA (BL#54127), *UAS-*DenMark (BL#33061), *5-HT1A*^Δ5kb^ (BL#27640), *5-HT1B*^MB05181^ (BL#24240), *5-HT2A*^MI00459^ (BL#31012), and *5-HT7*^MB01344^ (BL#23066); *UAS*-*d5HT1A*^*RNAi*^ (VDRC#106094), *UAS-d5HT1B*^*RNAi*^ (BL#27634), *UAS*-5-HT2A-RNAi (BL #31882); *UAS*-5-HT7-RNAi (BL#27273). The strains were backcrossed for at least five generations with *w*^*1118*^ (Scholz lab) to isogenize the genetic background. To insure that the transgene controls are similar to the experimental flies in the behavioral experiments, flies carrying the Gal4 or UAS transgenes were crossed to the *w*^*1118*^ background and the F1 generation carrying only one copy of a given transgene in comparison to the experimental groups were analyzed. In the S1 Table summarizes the stocks used with references.

The animal studies including the model organism Drosophila melanogaster were conducted in agreement with the regulations of the DFG and the Land North Rhine-Westphalia.

### Generation of *UAS*-Sert::GFP Transgene

To generate the *UAS*-Sert::GFP transgene the *Sert* cDNA was amplified from the RE10485 vector using the following linker primers GTATTTGCGGCCGCATGGACCGCAGCGG and GAATTAGGTACCCACCGAGGTGCCCTGT. The fragment was cloned into the UAS-eGFP vector via NotI and KpnI restriction sites and confirmed by sequencing analysis. The transgenes were generated by injecting the construct into *w*^*1118*^ embryos according standard procedures.

### Odor attraction

Olfactory attraction was determined as described before [6]. Briefly, approximately 50 3- to 6-day-old male flies were given the choice between two odor traps at 25°C and 60% relative humidity overnight. Only flies of one sex were used to avoid differences in behavioral response due to sexual dimorphism. After 16 h, the fly number in each odor trap was determined. A preference index (PI) was calculated as follows: PI = (#A—#_B_) / (#_A_ + #_B_), where #_A_ and #_B_ indicate the fly numbers in trap A and B, respectively. If more than 10% of flies did not enter any odor traps, the trial was not considered. The PI values ranged from 1 and—1. A positive PI indicates attraction and a negative aversion. The odor traps were filled with apple mango juice (Alnatura, Germany GTIN: 4104420071841). One odor trap was additionally filled with one of the following odorants: acetic acid (AA; VWR, Germany #20104.298), ethyl acetate (EtOAc; AppliChem, #A0681) or ethanol (EtOH; VWR, Germany #20821.321). Odor attraction assay paired with the opto-genetic set up was used as previously described [5].

### Negative geotaxis

The climbing abilities and negative geotaxis of flies were analyzed in a modified counter-current assay [33]. A group of 30 starved or fed 3- to 5-day-old male flies was inserted into tube #1 and mechanically knocked to the bottom. Flies were allowed to climb up for 30 s. Top flies were transferred into the second vial by moving the upper part of the vials. The procedure was repeated four times until the flies were inserted into the last vial. The number of flies in each tube was determined. Flies in the first two tubes were defined as group one, those in the third and fourth tube as group two, and those in tube five and six as group three. The relative number of flies in each group was calculated by dividing the number of each group by the total fly number. The control group and experimental group were tested in parallel.

### Olfactory learning and memory

The associative olfactory short-term learning and memory of the flies were assessed using a modified Tully and Quinn paradigm [34], [35]. Briefly, 100 three- to five-day-old male and female flies were starved for 16–20 h. Next, they were exposed to the first odorant in a tube containing a filter paper soaked in water for 2 min, followed by a 1-min long air exposure. The flies were then moved to a new tube where the second odorant was paired with the reinforcer of 2 M sucrose for 2 min. For aversive learning and memory, the exposure to the second odorant was paired with a negative reinforcer of a 1-min long electric shock (12 1.3-s pulses at 90 V spaced in 5-s intervals). After the training cycle for classical aversive learning, the flies were given a 1.5-min rest and retrained with the same odorants with reversal of the reinforcer to test aversive reversal learning [36]. After a 3-min rest, flies were given a choice between the reinforced and non-reinforced odorants for 2 min. Performance index (PI) was calculated as follows: PI = (#_cs+_- #_cs-_)/ (#_cs+_ + #_cs-_), where #_cs+_ and #_cs-_ indicate the fly numbers in the odor with the reward or punishment (conditioned odorant: CS+) and other odor (unconditioned odorant: CS-), respectively. To rule out non-associative effects, each experiment consisted of two reciprocal groups in which the punished or rewarded odors were switched. A single PI value reflected the average score of both PIs and was determined as follows: PI = (PI_1_ + PI_2_)/2. The odorants used for the conditioned stimulus (CS) were 3-octanol (3-OCT; 1:80 diluted in paraffin oil) and 4-methylcyclohexanol (MCH; 1:100 diluted in paraffin oil). The assay was performed at 25°C and 80% humidity. To test for odorant sensitivity and odorant balance, fed or starved flies were allowed to choose between paraffin oil and 3-OCT (1:80) or MCH (1:100) for 2 min (S2 Table). The electric shock reactivity of fed flies was tested by providing 12 electric shock of 90 V with a 1.3-s duration in 5-s intervals (S2 Table). Flies were then allowed to choose between a tube in which they received the electric shock and a neutral tube. To analyze sucrose perception, starved flies were placed in vertical tubes with a stripe of filter paper (width, 10 mm) soaked in either 2 M sucrose or water (S2 Table). After flies began to touch the paper, the time spent on the filter paper during a total period of 30 s was recorded. The response index (RI) was calculated by the time spent on the filter paper divided by 30 s [35].

### Food consumption

Food intake was measured using a modified capillary feeder (CAFE) assay [37], [38]. The food consisted of 5% sucrose (AppliChem, #A2211.1000), 5% yeast extract (Sigma-Aldrich; #70161-500G) and 2% food color (Ruth, Cochineal #E124). To measure food intake in fed flies fed ad libitum, a group of 8 three- to five-day-old male flies had excess to food for 24 h. To measure food intake of starved flies, a group of 20 male flies were starved for 18 h at 25°C and 60% relative humidity and then were fed for 3 h. The total food intake and the number of flies were determined. The food intake (μL/ fly) was calculated as the total food intake volume divided by the number of flies.

### Immunohistochemistry

Adult brains were labeled with primary and secondary antibodies after Schneider *et al*., 2012. The following primary antibodies were used: chicken anti-GFP (1:1000, Life Technologies), mouse anti-GFP (1:200, NeuroMab, clone N86/38), rat anti-5-HT (1:100, Millipore), mouse anti-nc82 (1:20, DSHB), rabbit anti-mCherry (1:500, Clontech), mouse anti-ChAT (1:100, DSHB), rabbit anti-GABA (1:100, Sigma), and rabbit anti-DvGluT (1:1000, kindly provided by Hermann Aberle [39]. The secondary antibodies included the following: Alexa Fluor 488 goat anti-chicken IgG (1:1000, Life Technologies), Alexa Fluor 488 goat anti-mouse IgG (1:200, Life technologies), Cy3 goat anti-rat IgG (1:200, Jackson Immunoresearch), Cy3 goat anti-rabbit IgG (1:200, Jackson Immunoresearch), and Cy3 goat anti-mouse IgG (1:200, Jackson Immunoresearch). Confocal stacks with 1-μm-thick optical sections were obtained using the Zeiss 510 confocal microscope and analyzed using Fiji software. Images were presented using Adobe Photoshop CS6.

### Statistics

The behavioral data were displayed as the mean ± s.e.m. The nonparametric one-sample sign test was used to test for significant differences from random choice. To determine whether there was a significant difference between more than two groups, one-way ANOVA with post hoc Tukey-Kramer was used. Statistical analyses were performed with StatView 5.0.1 (SAS Institute, Cary, NC, UAS) and Statistica 9.1 (StatSoft, Tulsa, OK, UAS).

## Results

Altered serotonin signaling in CSD neurons by blocking serotonin reuptake with the expression of a serotonin transporter with mutated serotonin binding sites increased the attractiveness of higher ethanol concentrations within a food odor blend [18]. Here, we wanted to analyze whether the attraction to other key odorants such as AA and EtOAc is also modified by CSD neurons.

### Serotonin transporter-dependent regulation of key odorant attraction

**Fig 1:**
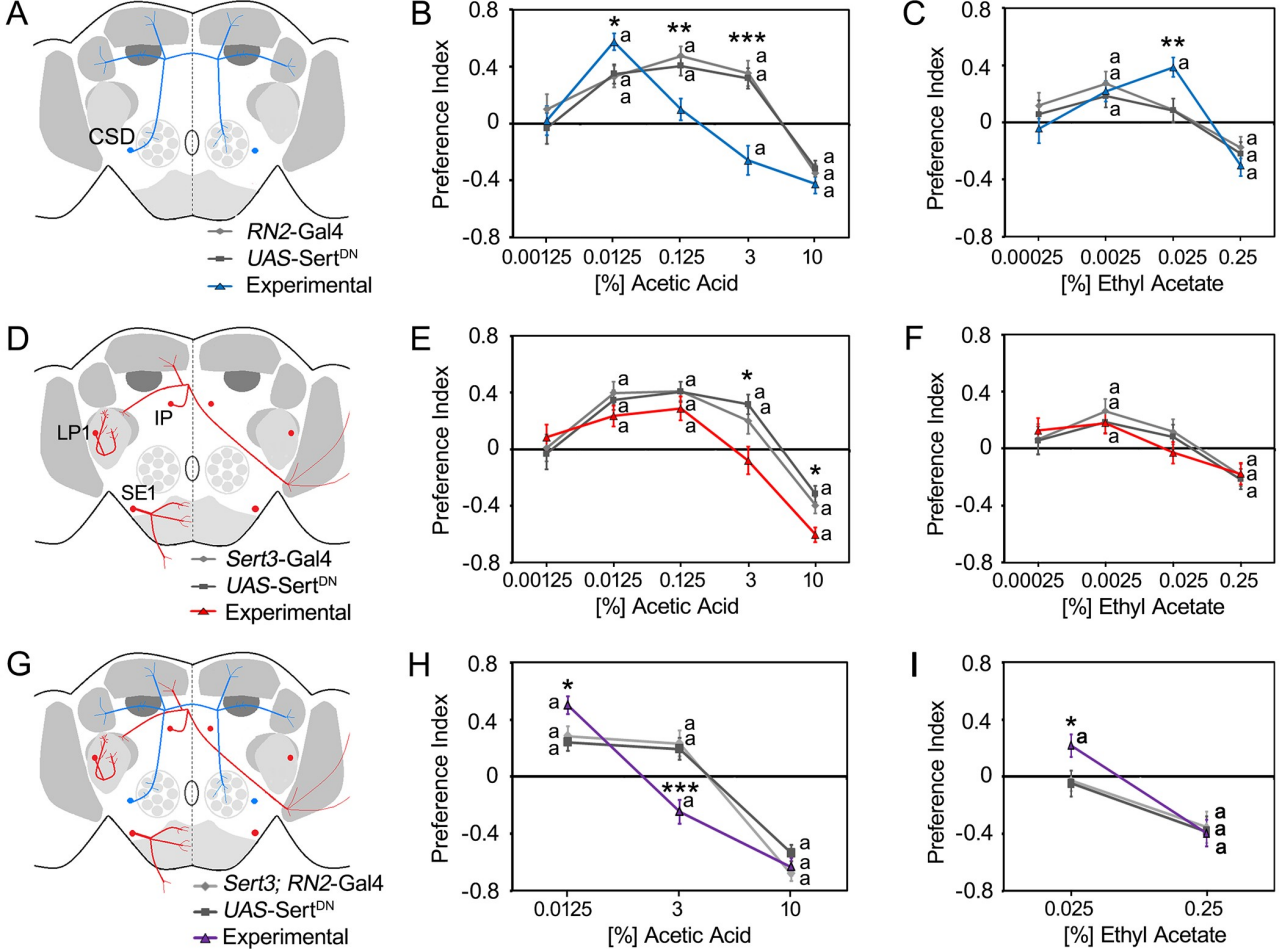
Key odorant attraction regulated by altered serotonin signaling. **A,** Schemata of the CSD neurons (blue lines) targeted by *RN2*-Gal4 (D) of the LP1, IP, and SE1 neuron clusters (red lines) targeted by the *Sert3*-Gal4 and (G) neurons targeted by both drivers are shown. **B**, Expression of the *UAS*-Sert^DN^ transgene under control of the *RN2*-Gal4 line significantly increased the attraction to 0.0125% acetic acid (AA) mixed with food odors and inhibited or even reversed the preference for 0.125% and 3% AA (N = 15–36). **C**, The expression significantly increased the attraction to 0.025% ethyl acetate (EtOAc)-enriched food odors (N = 17–32). **E**, Expression of *UAS*-Sert^DN^ in a *Sert3*-Gal4-dependent manner significantly inhibited the attraction to 3% AA and increased the aversion to 10% AA within food odor blends (N = 16–33). **F**, The expression did not significantly affect the behavioral response to EtOAc (N = 16–30). **H**, The expression of the mutant SERT protein under the control of the *Sert3*-Gal4; *RN2*-Gal4 drivers significantly increased attraction to 0.0125% AA but significantly reversed the attraction to 3% AA in food odors to aversion (N = 22–29). **I,** The behavioral attraction to 0.025% EtOAc was significantly increased in the experimental group (N = 27–32). A significant difference from random choice is labeled with the letter **a** as determined by one-sample sign test (*P* < 0.05). The stars indicate a significant difference between the experimental and two control groups as determined by ANOVA with post hoc Tukey-Kramer analysis with *P < 0.05, **P < 0.01, and ***P < 0.001. For the underlying numerical data see S3 Table.

To analyze whether serotonergic neurons regulate the attraction to key odorants in complex food odor blends, we used a binary choice assay consisting of two odor traps [6]. Both odor traps contained apple mango juice as a food odor source. In addition, one of the traps contained different concentrations of the odorant AA or EtOAc. Both odorants function as key odorants in food odor blends [19], [40], [41]. To alter serotonin signaling selectively, we expressed a mutated version of the serotonin transporter that fails to bind serotonin, the *UAS*-Sert^DN^ transgene, in CSD neurons using the *RN2*-Gal4 driver (Fig 1). The expression of *UAS*-Sert^DN^ reduces presynaptic serotonin levels, a phenotype that is expected when the reuptake of secreted serotonin from the synaptic cleft into the presynaptic neuron is blocked [18]. The expression of a mutated serotonin transporter interferes in serotonin signaling however the tool does not allow determining when proper serotonin signaling is acutely required to regulate odorant attraction.

In controls, food odors containing a low concentration of AA and EtOAc were significantly more attractive than plain food odors, whereas food odors containing a higher concentration were significantly more aversive (Fig 1B and 1C). Expression of mutated serotonin transporter in CSD neurons significantly enhanced the attraction to low-concentration (0.0125%) AA and significantly increased the attraction to higher-concentration (0.025%) EtOAc-containing food odor mixtures but did not alter the attraction to low EtOAc concentrations (Fig 1A–1C). Thus, altered serotonin transporter function in CSD neurons affects the attraction of odorants in an odorant-specific manner. The observed increase in attraction to higher concentration of EtOAc is similar to the observed attraction of flies with altered serotonin transporter signaling in CSD neurons to a median concentration of ethanol [18]. Increased serotonin signaling using optogenetics and activation of a second set of serotonergic neurons reduces the attractiveness of the key odorant ethanol within food odor blends via behavioral inhibition. These set includes neurons of the LP1, IP, and SE1 clusters that do not directly innervate the olfactory pathway [18].

We next addressed whether the inhibition of attraction is odorant specific. Therefore, we alter serotonin signaling in the neurons of the LP1, IP and SE1 cluster by expressing the *UAS*-Sert^DN^ transgene under the control of the *Sert3*-Gal4 driver (Fig 1D–1F) and analyzed the consequences on odor trap choice of the flies. Expression of a mutated serotonin transporter in *Sert3*-Gal4-targeted neurons blocked the attraction to 3% AA and increased the aversion to 10% AA compared to controls (Fig 1E). This alteration did not significantly affect EtOAc attraction, although the attraction to 0.025% EtOAc decreased slightly (Fig 1F). Thus, serotonergic inhibition of a second set of serotonergic neurons including the LP1, IP, and SE1 neurons effects the attraction to AA similarly to the attraction to ethanol. To test whether behavioral inhibition influences the odorant attraction or vice versa, we analyzed the attraction to AA and EtOAc in flies with altered serotonin signaling in CSD and LP1, IP, and SE1 neurons by combining the RN2-Gal4 and *Sert3*-Gal4 drivers (Fig 1G–1I). In these flies the attraction to 0.125% AA within food odor blends was significantly increased, and in control flies the attraction to 3% AA observed was reversed to aversion. The behavioral response to 10% AA was not altered (Fig 1H). In addition, the attraction to 0.025% EtOAc in food odor blends was enhanced, but the aversion to 0.25% EtOAc was unaffected (Fig 1I). The behavioral responses were similar to those observed when serotonin transporter expression was only altered in CSD neurons. Therefore, when serotonin transporter function is altered in the CSD neurons and LP1, IP and SE1 neurons, the behavioral outcome is dominantly regulated by the serotonergic CSD neurons.

**Fig 2:**
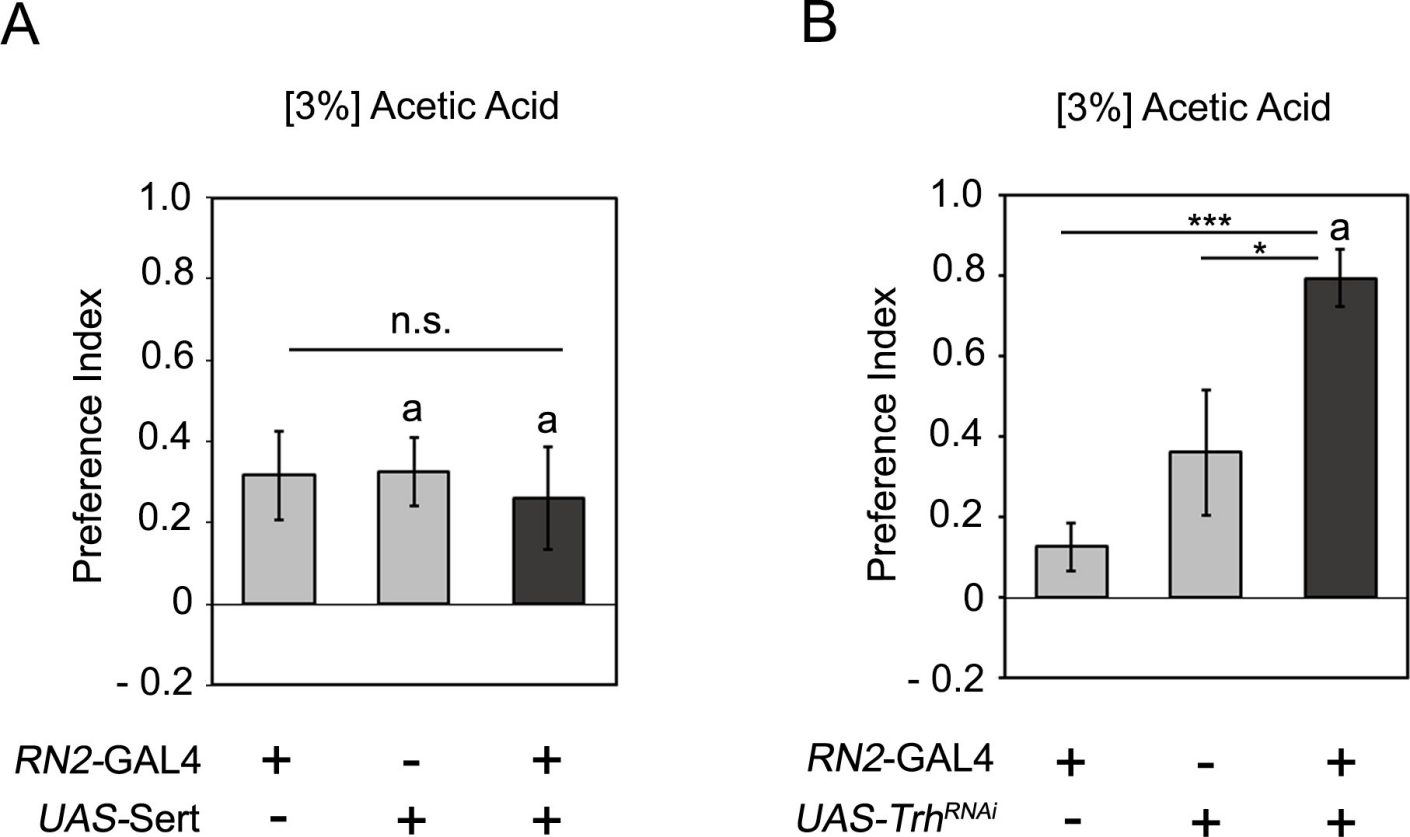
Serotonin acts as negative regulator in key odorant attraction. **A,** Overexpressed of the serotonin transporter in the CSD neurons did not change attraction to 3% AA containing food odors. (N = 11, 18, 18). **B**, The RNAi mediated knock down of *Trh* significantly increases the attraction to the 3% AA containing food odor (N = 10, 18, 14). Differences from random choice were determined by one-sample sign test and significant differences were labeled with the letter a (*P* < 0.05). The ANOVA with post hoc Tukey-Kramer analysis was used to determine significant differences between the experimental and two control groups with *P < 0.05, **P < 0.01, and ***P < 0.001. For the underlying numerical data see S4 Table.

To investigate whether the reduced attraction to 3% AA in the CSD neurons is due to reduced or increased serotonin signaling, we overexpressed the serotonin transporter under the control of the *RN2*-Gal4 driver and analyzed the attraction of 3% AA containing food odors (Fig 2A). More serotonin transporter should increase serotonin uptake from the synaptic cleft and thereby reducing serotonin signaling in the synaptic cleft. The attraction to 3% AA did not differ between experimental and control animals (Fig 2A). To investigate whether serotonin is required to regulate odorant attraction to 3% AA, we eliminated serotonin expression in the CSD neurons by RNAi mediated know down of the tryptophan hydroxylase (Trh) enzyme, an enzyme required for serotonin synthesis. The expression of the *Trh*-RNAi transgene eliminates serotonin expression [28]. The RNAi mediated knock down of serotonin in CSD neurons significantly increased the attractiveness to 3% AA containing food odors (Fig 2B). Thus serotonin is required as negative regulator for 3% AA odorant attraction. Together with the observation that the expression of the SertDN transgene reduced odorant attraction, these results are consistent with the function of SertDN in blocking serotonin uptake and increasing serotonin signaling.

To investigate whether the observed difference in the attraction might be due to possible motor defects, we analyzed the climbing behavior of flies with altered serotonin signaling in CSD neurons or LP1, IP, and SE1 neurons in the counter-current assay (S1 Fig). Altered serotonin signaling in neither set of neurons interfered with the abilities to climb of starved flies or flies fed ad libitum; therefore, motor defects unlikely influenced the choice between the two odor traps. Thus, increased serotonin signaling in CSD neurons can enhance the attraction to a low concentration of different key odorants or alternatively enhance the attractiveness of higher concentrations of key odorants that are normally less attractive.

### Cell-cell contacts of the CSD neurons onto the LNs, PNs and Kenyon cells of the MBs

**Fig 3:**
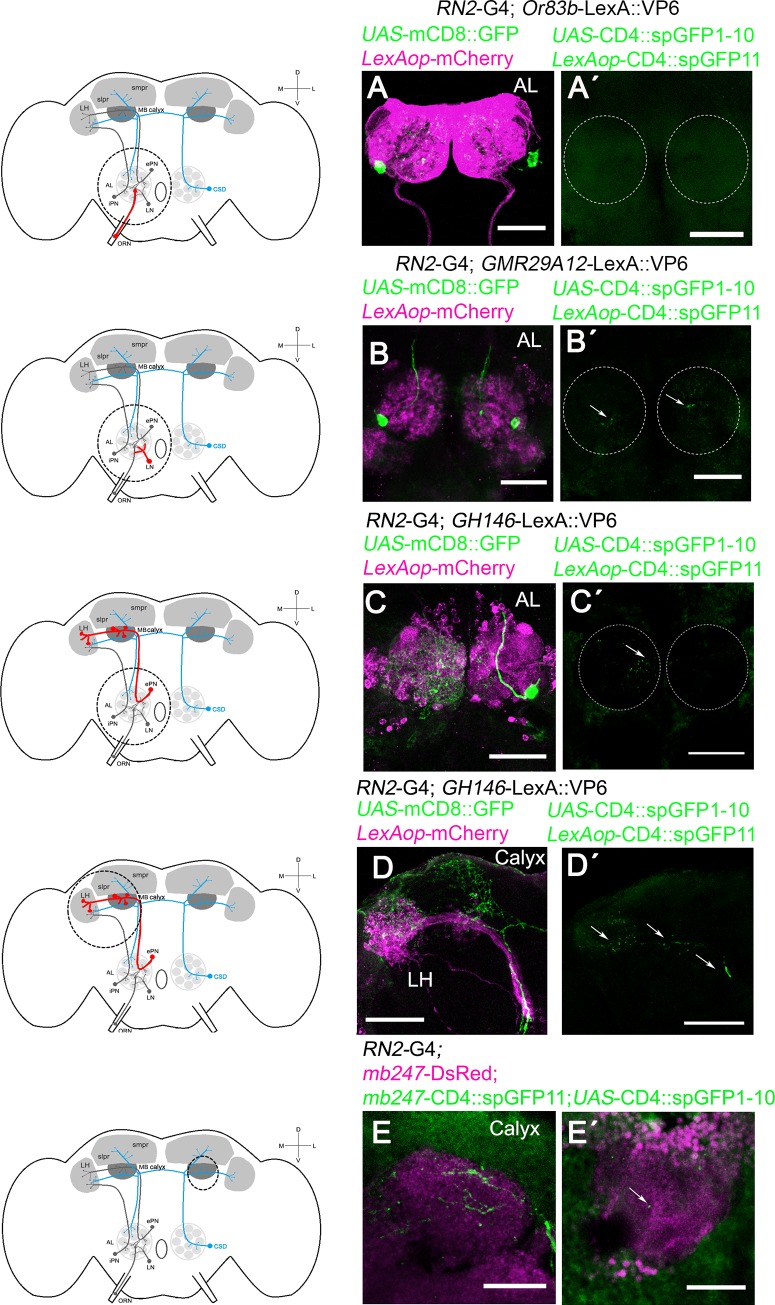
The connections between CSD neurons and LNs, PNs, or Kenyon cells. A schema is provided indicating the region of interest with a circle, the CSD neuron in blue and neurons of interest in red. The dots indicate that a second set of neurons exist in opposite brain hemisphere. **A-D,** the CSD neurons are labeled using *UAS*-mCD8::GFP under the control of *RN2*-GAL4 in green. The expression pattern of the, **A**, *Or83b*-LexA::VP16 driver targeting the ORNs, **B**, *GMR29A12*-LexA targeting the LNs and, **C-D**, the *GH146*-LexA targeting the PNs with projections in the AL, LH and MBs are visualized using the *LexAop*-myr::mCherry transgene (in magenta). In **A´-E´,** *RN2*-GAL4 drives the expression of the *UAS*-CD4::spGFP1-10 transgene and, in **A´-D´**, LexA drives the expression of the LexAop-CD4::spGFP11 transgenes. In **E** and **E´**, the *mb247*-DsRed; *mb247*-CD4::spGFP11 transgenes label the MBs in magenta, and spGFP11 is expressed under the control of the MB-specific promotor *mb247*. In E GFP expression targeted by the RN2- driver and *mb247*-DsRed are shown. In **B´**- **E´**, the reconstituted GFP is seen as green dots. Dashed circle indicates the approximate position of ALs, and the arrow in **B´-E´** indicates the reconstituted GFP signal. Scale bar is 50 μm in **A—D´** and 25 μm in **E** and **E´**.

To better understand how serotonin in the CSD neurons influences the odorant attraction, we analyzed the morphology of the CSD neuron. The CSD neurons projected to the ipsilateral AL, the calyx of the MBs, and the LH and terminate in the contralateral AL [13], [12] (S2 Fig). We next analyzed the projection pattern of the CSD neurons in relationship to their putative target/interacting neurons in the olfactory pathway including the olfactory receptor neurons ORNs and the neurons of the LNs, the PNs and the MBs (Fig 3). Therefore, we combined the *UAS*/Gal4 with the LexA/LexAop system or a marker line for the MBs. CSD neuron projections were visualized by the *UAS*-mCD8::GFP transgene under the control of the *RN2*-Gal4 driver. To visualize the projection of the ORNs, LNs, LH and MBs, we used the *LexAop*-myr::mCherry transgene under the control of *Or83b*-LexA::VP16, *GMR29A12*-LexA, *GH146*-LexA and *mb247*-DsRed, respectively. We confirmed whether the neuron forms cell-cell contacts using the GFP reconstitution across synaptic partners (GRASP) technique [25]. Previously it has been shown that the ORNs innervating the DL4, DM2 and DM1 form presynaptic contacts with the CSD neuron using the same *Or83b*-LexA::VP16 driver and a syb:GRAPspGFP1-10 transgene in combination with the MB465-sp-Gal4 driver and the CD4-spGFP transgene [42]. The ORNs targeted by the *Or83*-LexA driver project in close vicinity to the CSD neurons in the AL (Fig 3A). In contrast to previous results we did not find cell-cell contacts (Fig 3A’) between the ORNs targeted by the *Or83*-LexA driver and the CSD neurons. This might be explained by differences in the environmental interaction and/or the reproductive state of the animals that influence neuronal plasticity at the level of the ALs, since the expression of distinct subgroups of chemoreceptors depends on the reproductive state and previous environmental interactions [43]. Thus under our growing conditions Or83 positive neurons in male flies do not appear to form cell/cell contacts with the CSD neurons directly. The CSD neurons are in close proximity to the LNs targeted with *GMR29A12-LexA* and form cell-cell contacts with the LNs as revealed by GRASP analysis (Fig 3B and 3B´). The PNs targeted with the *GH146*-LexA driver project and form cell-cell contacts with the CSD neurons in the AL, in the region of the calyx of the MBs and the LH (Fig 3C and 3C´; Fig 3D and 3D´). In addition, direct cell-cell contacts of the CSD neuron with the calyx of the MBs were observed as the reconstituted GFP signal was detected in flies carrying the RN2-Gal4 driver in combination with the *mb247*-Ds2Red, *mb247*-CD4::spGPF11, and *UAS*-CD4::GFP1-10 transgenes (Fig 3E and 3E´). These observations support previous finding showing serotonin and serotonin transporter expression at the calyx of the mushroom bodies [19]. In addition, serotonergic neurons targeted by the *Trh*-Gal4 driver that includes the serotonergic CSD neurons also exhibited connections with Kenyon cells in the calyx of the MBs [24]. The CSD neurons receive and provide input at the site of the AL [44] and synapse onto LNs and PNs in the AL [42]. To analyze whether the CSD neurons might receive input or give input to the LNs, PNs or Kenyon cells, the expression patterns of postsynaptic and presynaptic markers in CSD neurons were analyzed using the postsynaptic marker, Denmark [45] and presynaptic protein Bruchpilot BRP; [23]; (S2B–S2E Fig). The expression of the UAS-Brp transgene and UAS-Denmark in CSD neurons are found at the sites of the AL. Thus consistent with previous results the CSD neurons might receive and provide input at the sites of the AL [44]. In addition, CSD neurons directly connect with the calyx of the MBs and indirectly via PNs at the site of the calyx.

**Fig 4:**
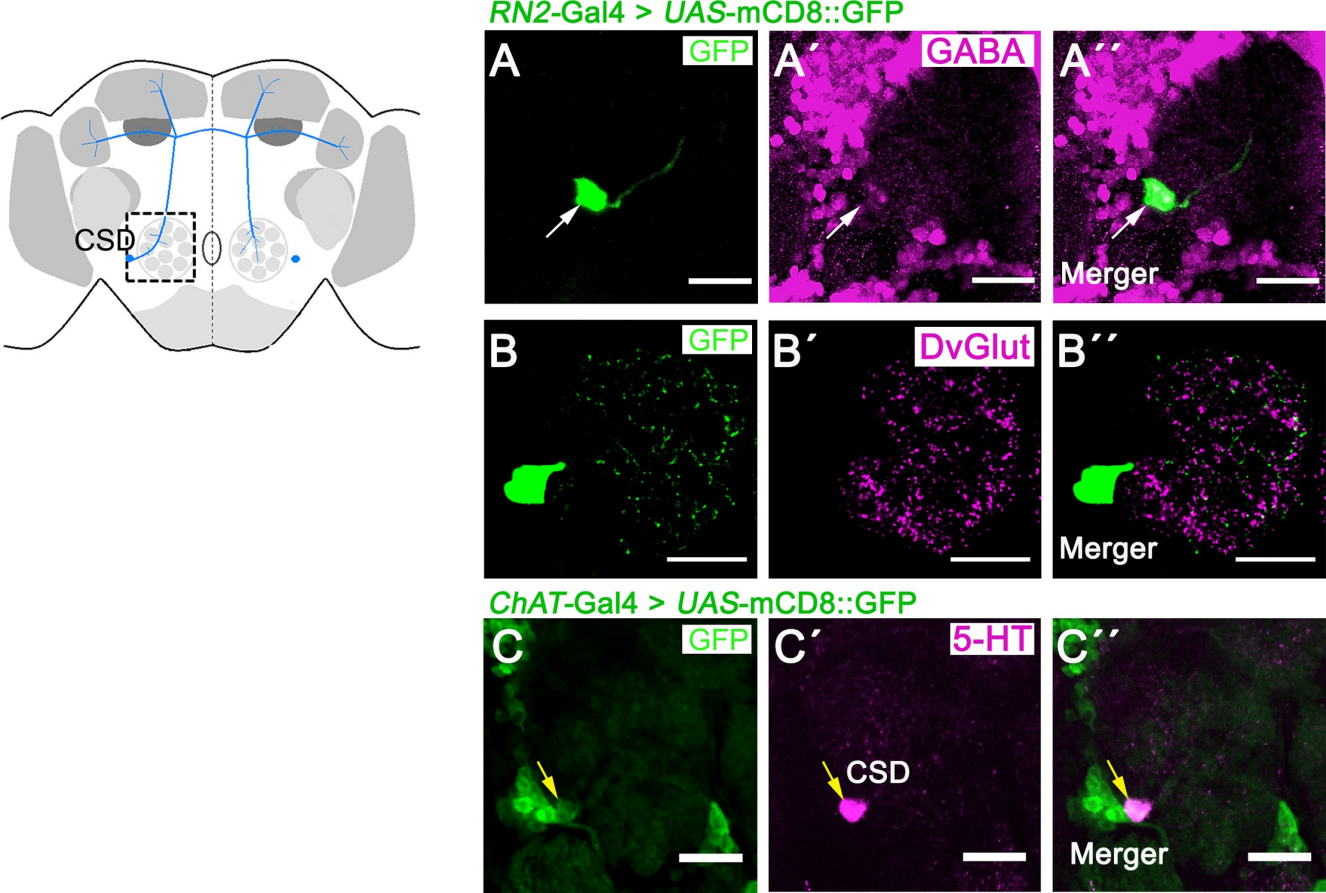
No co-localization of markers for GABA or glutamate in CSD neurons. A schema is provided indicating the region of interest–the antennal lobes- with a square. In **A—C,** the expression pattern of the *RN2*-Gal4 driver targeting CSD neurons are visualized using *UAS*-mCD8::GFP (in green). **A—A´´**, the cell bodies of CSD neurons (green) do not colocalize with GABA staining (in magenta) or, in the region of the ALs, **B—B´´**, with DvGlut (in magenta). **C—C´´**, the cholinergic neurons targeted by the *ChAT*-Gal4 driver (in green) co-express serotonin 5-HT (in magenta). **A—C** shows a representative 1-μm-thick slice of a confocal stack. The white arrow in **A—A´´** indicates the position of CSD neurons in the adult brain, and the yellow arrows in **C** indicate co- expression of respective markers. Scale bars represent 25 μm.

Recently, the *ChAT*-Gal4 driver has been shown to label CSD neurons and those positive for the choline acetyltransferase (ChAT) and the vesicular acetylcholine transporter (VAchT) expression [44]. To investigate whether the CSD neuron expresses GABA or glutamate, we performed co-localization studies. Therefore, the Gal4 expression domain of the RN2-Gal4 driver was visualized using the *UAS*-mCD8::GFP transgene in combination with antibodies against GABA and the vesicular glutamate transporter (DvGlut) as marker for glutamate (Fig 4A to 4B). CSD neurons did not express GABA or DvGlut. Consistent with previous results, the *ChAT*-Gal4 driver also expresses Gal4 in a serotonergic neuron at the antennal lobes (Fig 4C; [44]). Thus, the CSD neurons do not express GABA or glutamate.

### No interference in olfactory learning and memory or food consumption by altered serotonin signaling in the CSD neurons

**Fig 5:**
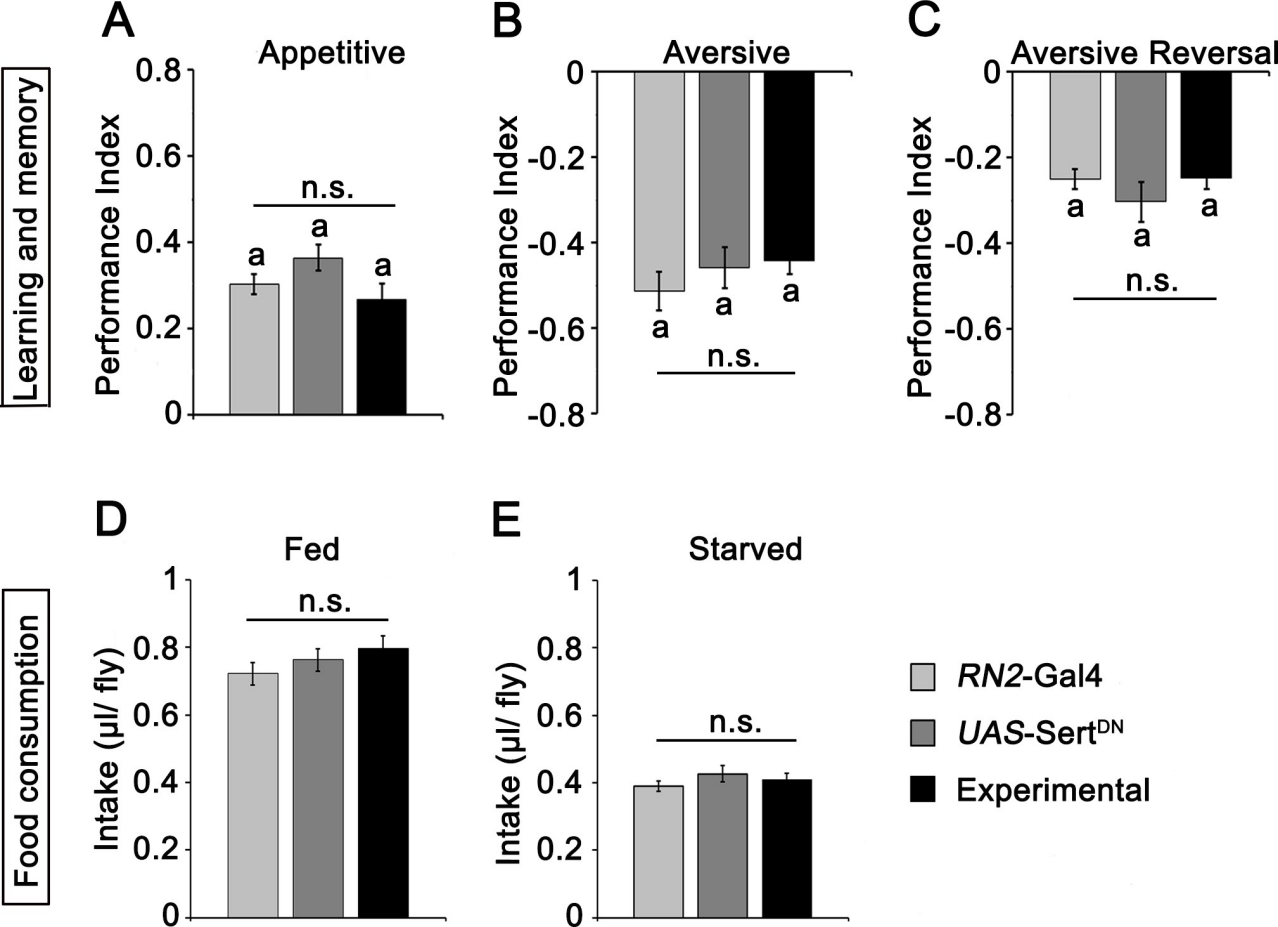
No function of CSD neurons in olfactory learning and memory or food intake. **A** and **B**, olfactory appetitive and aversive learning and 2-min memory were not affected when serotonin signaling was prolonged in CSD neurons by expression of *UAS*-Sert^DN^ (N = 8–12). In **C**, prolonged serotonin signaling did not influence aversive reversal learning and memory (N = 8) or, in fed, **D**, or starved flies, **E**, the intake of 5% sucrose and 5% yeast food solution (N = 15–25). Non-significant differences are labeled with **n. s.** (*P* > 0.05), and the letter **a** indicates significant differences from random choice as determined by one-sample sign test (*P* < 0.05). For the underlying numerical data see S5 Table.

The CSD neurons form synapses with the calyx of the MBs (Fig 3E´). The MB is the major structure involved in olfactory learning and memory [46]. To test whether serotonergic signaling in the CSD neurons influences learning and memory, we tested flies with increased serotonin signaling in the CSD neurons in three different olfactory short-term learning and memory paradigms, including appetitive, aversive, and aversive reversal learning and memory by using the Tully Quinn paradigm (Fig 5). Flies with altered serotonin signaling in the CSD neurons showed normal odorant perception to 3-OCT and MCH, normal preference for 2 M sucrose, and normal sensitivity to a 90-V shock (S2 Table). The flies with altered CSD neuron serotonin signaling learned and remembered the pairing of the odorants 3-OCT or MCH with 2 M sucrose as a positive reinforcer or a 90-V electric shock as negative reinforcer 3 min after the training equally well as controls (Fig 5A and 5B). We have previously suggested that serotonin release from the CSD neurons stabilizes odor information flow [18]. If CSD-derived signal is more stable and long-lasting, then this signal should persist at the mushroom bodies and, thus, may interfere with olfactory signal attenuation or the learning of a new reinforced odorant signal. We next investigated whether increased CSD signaling affects reversal learning by expressing the UAS-Sert^*DN*^ transgene under control of the *RN2*-Gal4 line and testing these flies in a reversal aversive olfactory learning and memory paradigm [36]. First, flies learned to associate the first odorant with electric shock; then, after 5 min, the flies were training with the second odorant paired with a 90-V electric shock for 1 min. The flies reversed their choice between the two odors and avoided the odor most recently paired with the electric shock after a single reversal training cycle in both the control and experimental groups (Fig 5C). Starvation promotes appetitive olfactory behavior by enhancing sensitivity to attractive odorants and reducing sensitivity to aversive odorants within the context of food-related odors [47] [48]. However, the innate odorant response to MCH or 3-OCT is not significantly altered in hungry or flies fed ad libitum (S2 Table).

Serotonin might change appetitive behavior depending on the hungry or flies fed ad libitum and therefore indirectly changes the value of a reinforcer such as 2 M sucrose. To address whether serotonin signaling in CSD neurons influences food consumption, food intake was tested using the CAFE assay in fed and starved flies (Fig 5D and 5E). Increased serotonergic signaling in CSD neurons did not affect food consumption in starved flies or flies fed ad libitum (Fig 5D and 5E). In summary, increased serotonin signaling in CSD neurons did not alter food intake, appetitive or aversive olfactory short-term learning and memory or odor reversal learning.

### Serotonin receptor modulation of the attraction to key odorants

**Fig 6:**
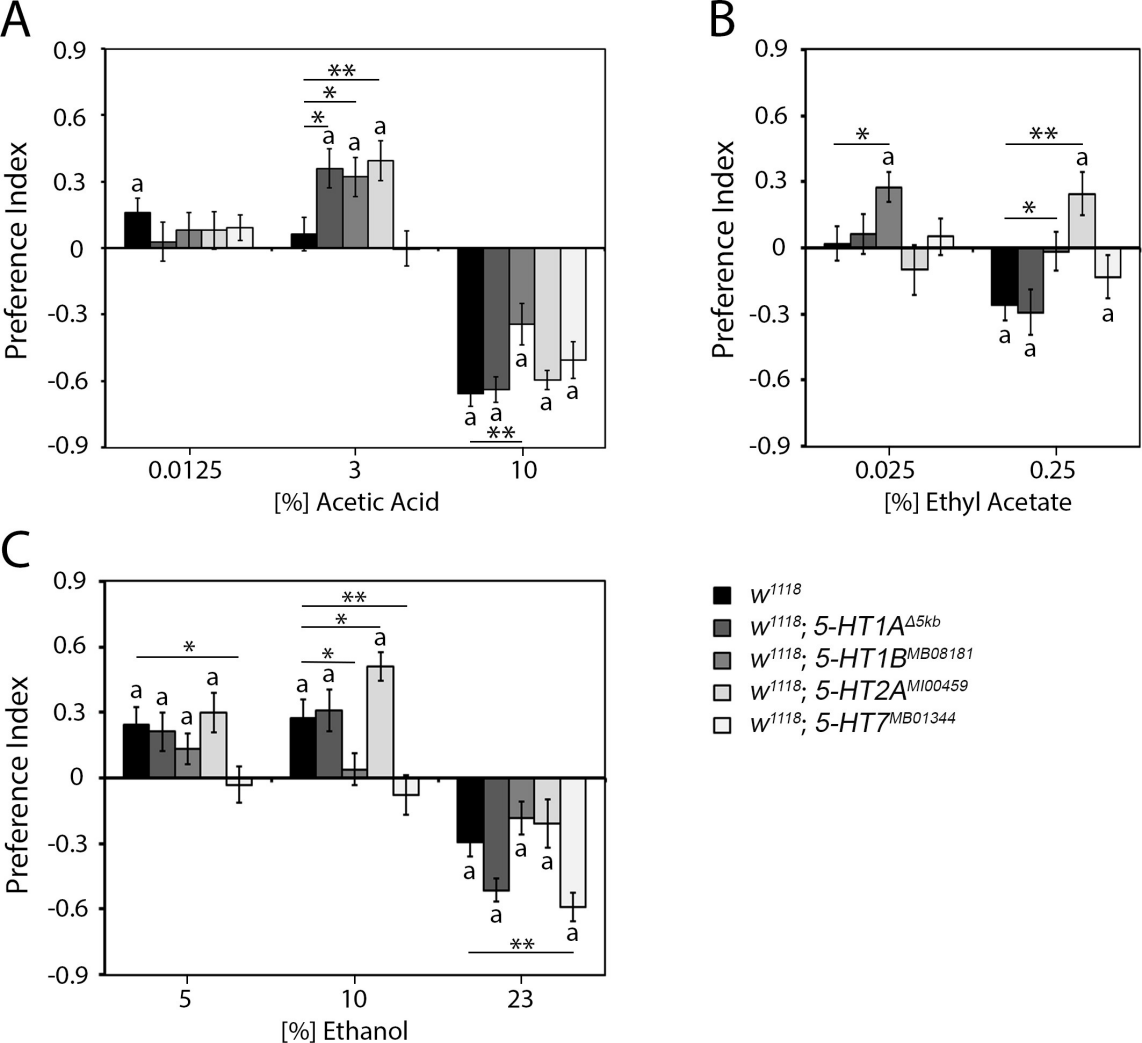
Regulation of odorant attraction by different serotonergic receptors. **A,** the *5-HT1A*^Δ5kb^, *5-HT1B*^*MB05181*^ and *5-HT2A*^*MI00459*^ receptor mutant flies were significantly more attracted to 3% AA-enriched food blends than the controls or *5-HT7*^*MB01344*^ mutant flies. The *5-HT1B*^*MB05181*^ flies found 10% AA-enriched food blends significantly less aversive than controls (N = 21–34). **B**, The *5-HT1B*^*MB05181*^ mutant flies exhibited significantly greater attraction to 0.025% EtOAc and showed no aversion to 0.25% EtOAc. The *5-HT2A*^*MI00459*^ mutants were significantly attracted to an otherwise aversive 0.25% EtOAc-enriched odor blend (N = 23–28). **C,** The *5-HT1B*^*MB0518*^ flies did not show significant attraction to 10% ethanol-enriched food blends. The *5-HT2A*^*MI00459*^ mutant flies were significantly more attracted to 10% ethanol-enriched food blends than the control flies. The *5-HT7*^*MB01344*^ flies were not attracted to any tested ethanol concentration and showed a significantly increased aversion to 23% ethanol compared to control flies (N = 24–31). Significant differences from random choice are labeled with the letter **a** as determined by one-sample sign test (*P* < 0.05). The stars indicate a significant difference as determined by one-way ANOVA followed by post hoc Tukey-Kramer analysis (P* < 0.05; P** < 0.01). For the underlying numerical data see S6 Table.

To better understand how serotonergic CSD neurons might on one hand increase odorant attraction to odorants such as 3% AA and on the other hand prolong the attraction to a higher concentration of odorants such as EtOAc and ethanol, we wanted to identify possible postsynaptic serotonin receptors that mediate the changes in odorant attraction. In the ALs, 5-HT receptors including 5-HT1A, 5-HT1B, 5-HT2A, and 5-HT7 are expressed in the PNs and distinct classes of LNs [22]. First, we analyzed the behavioral response of four serotonin receptor (5-HT1A, 5-HT1B, 5-HT2A, and 5-HT7) mutants to different odor choices (Fig 6). We used mutants with alter serotonin receptor function to screen for receptors that might be involved in the regulation of odorant guided behavior at the level of the olfactory pathway. We expect that altered receptor function should result in the opposite phenotype of altered Sert function in the CSD neurons that increase serotonin signaling in the synapse. Here, again, one odor trap was filled with food odor and the second trap with food odor enriched with different concentrations of the key odorant AA, EtOAc or ethanol.

The *5-HT1A*^Δ5kb^ flies did not differ in the attraction to different key odorants concentrations from the control *w*^*1118*^ flies, except for an enhanced attraction to 3% AA (Fig 6A–6C). The results suggest that 5-HT1A is important in the regulation of the attraction to 3% AA but not to EtOAc or ethanol. The *5-HT1B*^MB08181^ flies showed an enhanced attraction to 3% AA a similar to *5-HT1A*^Δ5kb^ mutants (Fig 6A). In contrast to the *5-HT1A*^Δ5kb^ mutants, the *5-HT1B*^MB05181^ flies showed a reduced aversion to a higher concentration of 10% AA, an increased attraction to 0.025% EtOAc, and a reduced aversion to a higher concentration of EtOAc (Fig 6B). Furthermore, these flies show a reduced attraction to 10% EtOH and a reduced aversion to 23% EtOH (Fig 6C). Compared with the attraction in controls, the attraction to 3% AA, 0.25% EtOAc and 10% EtOH were increased in the *5-HT2A*^*MI00459*^ mutants (Fig 6A–6C). The *5-HT7*^*MB01344*^ mutants did not differ from controls in the attraction to different AA or EtOAc concentrations within food odor blends (Fig 6A and 6B), but showed a significant loss of attraction to 5% and 10% EtOH and showed a higher aversive responsive to a 23% EtOH (Fig 6C).

**Fig 7:**
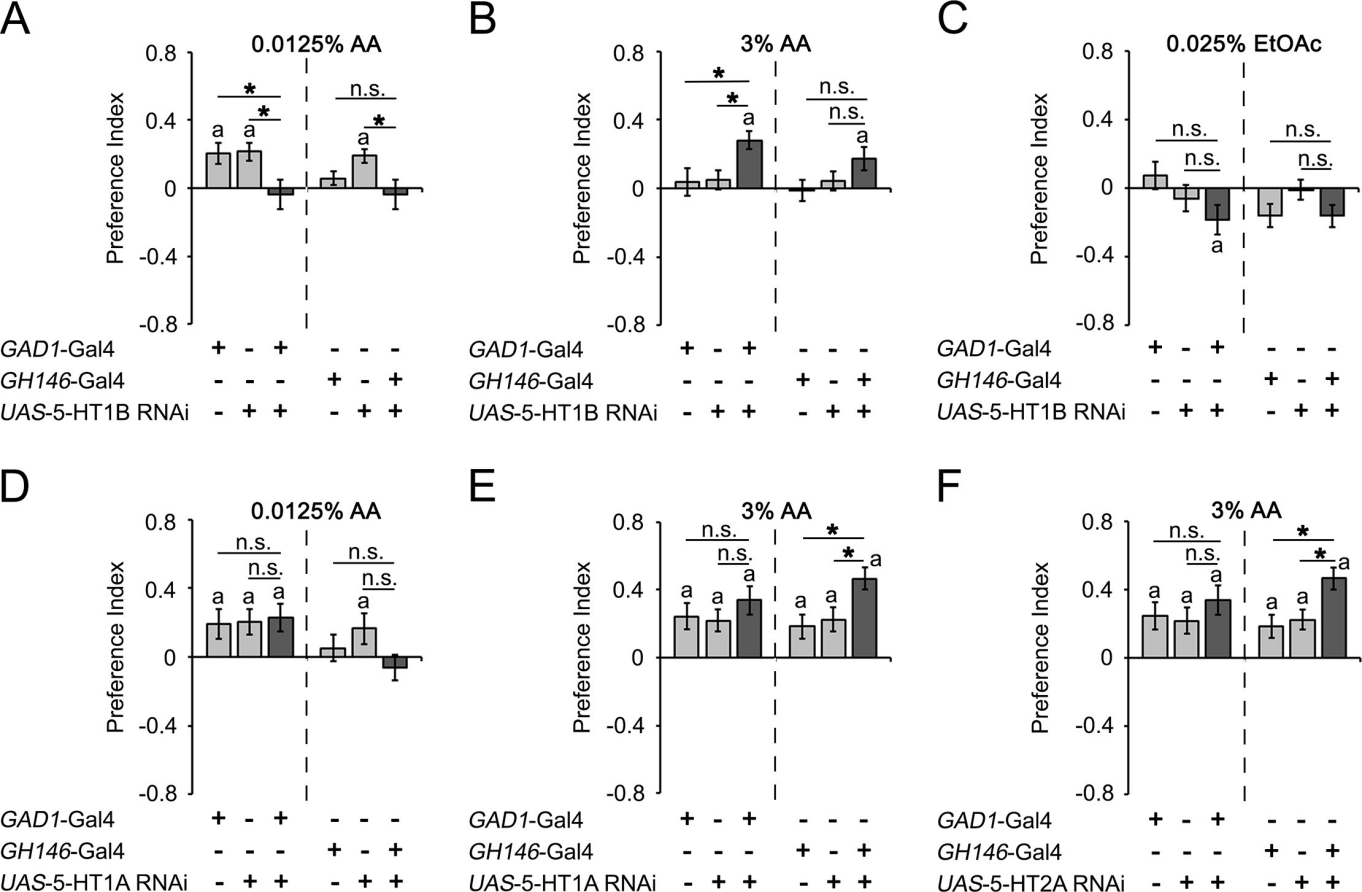
Differential function of 5-HT receptors in GABAergic neurons and PNs in odorant attraction. **A**, The expression of 5-HT1B RNAi in the LNs using the *GAD1*-Gal4 driver inhibited the attractive response to 0.0125% AA-enriched food odor blends, while 5-HT1B RNAi expressed in the PNs by using the GH146-Gal4 driver did not result in significant differences from the controls (N = 26–34). **B**, The attraction to 3% AA was increased by expression of 5-HT1B RNAi in the GABAergic neurons compared to the attraction in genetic controls (N = 30). **C**, The expression of 5-HT1B RNAi in the GABAergic neurons or PNs had no effect on 0.025% EtOAc attraction (N = 23–24). **D**, No differences in 0.0125% AA attraction was observed when the RNAi of 5-HT1A was expressed in the GABAergic or PNs compared to 0.0125% AA attraction in genetic controls (N = 24–26). **E—F**, The expression of 5-HT1A (**E**) or 5-HT2A (**F**) RNAi in the PNs induced a significant increase in 3% AA attraction compared to controls (N = 22–29). The star indicates a significant difference as determined by one-way ANOVA followed by post hoc Tukey-Kramer analysis (P* < 0.05), and the letter **a** indicates a significant difference from random choice as determined by One-sample sign test (*P* < 0.05). For the underlying numerical data see S7 Table.

To further explore how different serotonin receptors contribute to the odorant attraction, we analyzed the function of 5-HT1A, 5-HT1B and 5-HT2A receptors in PNs and GABAergic neurons including the LNs (Fig 7). We focused on these receptors since the mutants of these receptors showed differences in the attraction levels in response to key odorants rather than selective changes in aversion, such as the change observed in 5-HT7 receptor mutants. To alter receptor function, we knocked down receptor function using *UAS-RNAi* transgenes for the respective receptors in combination with the GABAergic neuron driver GAD1-Gal4 or the projection neuron driver GH146-Gal4 (Fig 7). The GAD1-Gal4 driver drives transgene expression in a broad set of GABAergic neurons including the lateral neurons [27], [49]. Loss of 5-HT1B function in GABAergic neurons significantly reduced the attraction to a low concentration of AA (0.0125% AA) and increased the attraction to a higher concentration of AA (3% AA), but loss of 5-HT1B function in the projection neurons did not significantly alter the attraction to low or high concentrations of AA compared to all controls (Fig 7A and 7B). The knockdown of 5-HT1B function in GABAergic neurons resulted in patterns that resembled the observed increased attraction to a higher concentration of AA in the 5-HT1B mutants, showing that 5-HT1B function is required in GABAergic neurons to regulate the attraction to AA (Fig 6A). In contrast, the knockdown of 5-HT1B function in projection neurons and GABAergic neurons did not change the attraction to EtOAc and did not resemble the *5-HT1B*^MB05181^ mutant phenotype of increased attraction and decreased aversion to low and high concentrations of EtOAc (Fig 7C and Fig 6B), showing that the 5-HT1B receptor is not required for EtOAc attraction regulation in projection or GABAergic neurons. Therefore, the function of the 5-HT1B receptor in the GABAergic neurons is odorant specific, and 5-HT1B acts as a positive regulator for low and as a negative regulator for higher concentrations of AA. Knockdown of 5-HT1A in the GABAergic neurons did not alter the attraction to low or higher concentrations of AA; however, loss of 5-HT1A receptor function in the projection neurons significantly increased the attraction to high concentrations of AA, resembling the phenotype of the *5-HT1A*^Δ5kb^ mutant (Fig 7D and 7E and Fig 6A). A similar behavior was observed when 5-HT2A was altered in PNs and GABAergic neurons (Fig 7F). Here, loss of function of 5-HT2A in GABAergic neurons did not affect the attraction, but 5-HT2A loss of function in PNs significantly enhanced the attraction. Again, the increased attraction resembled the *HT2A*^*MI00459*^ mutant phenotype (Fig 7F and Fig 6A).

Taken together, the results of the serotonin receptor mutant phenotypes and the selective knockdown of serotonin receptor function in the PNs and GABAergic neurons demonstrate that 5-HT1A and 5-HT2A in the projection neurons are required as negative regulators for higher concentrations of AA (3% AA), whereas 5-HT1B in GABAergic neurons are required as a positive regulator for low (0.0125%) and as negative regulator for higher concentrations of AA (3% AA).

### Neuronal activation and loss of evoked neurotransmitter release in the CSD neurons repress attraction

**Fig 8:**
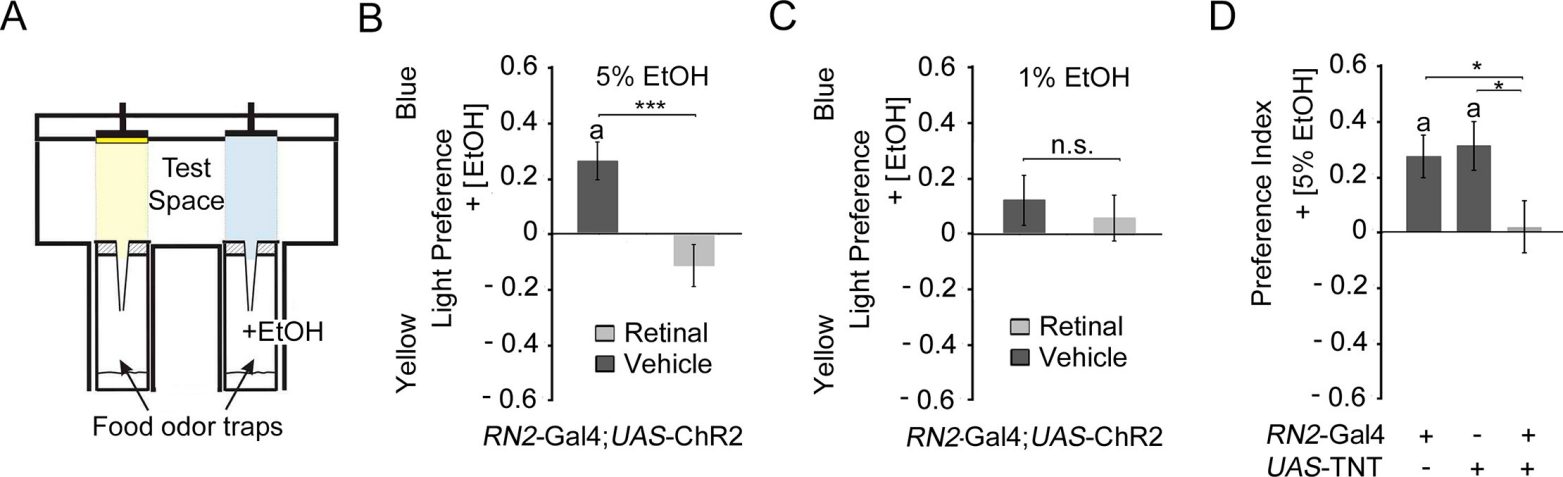
Activation and inhibition of CSD neurons result in loss of attraction to 5% EtOH. **A,** Schema of the binary odor trap assay paired with an opto-genetic set up (modified after Schneider et al., 2012). **B**, The activation of the CSD neurons using the *UAS*-ChR2 transgene under the control of the *RN2*-Gal4 driver inhibited the attraction to 5% EtOH-enriched food odor blends (N = 21; 23). **C**, The activation did not alter the indifference between a 1% EtOH-enriched food odor blend and the food odor blend (N = 17; 16). **D**, The attraction to 5% EtOH was significantly blocked by expression of *UAS*-TNT transgene in the CSD neurons compared to the transgene controls (N = 21; 21; 21). The Student`s *T*-test was used to determine differences between two groups and the one-way ANOVA followed by post hoc Turkey-Cramer analysis was performed for more than two groups. The stars indicate significant difference as determined by (P* < 0.05; P** < 0.01). The letters **n. s.** label non-significant differences and the letter **a** significant difference from random choice as determined by One-sample sign test. The errors are s.e.m. For the underlying numerical data see S8 Table.

To address whether the serotonin transporter function in the CSD neurons is required in the adult fly to regulate odorant attraction and whether the serotonin transporter acts in a similar manner in the CSD neurons as in the Sert3-Gal4 targeted neurons, we activated the neurons targeted by the CSD neuron using channel rhodopsin and analyzed the response to the key odorant enriched food sources (Fig 8). We used ethanol, since it is a key odorant and we wanted to compare the results with the recently published observation of the function of the serotonin transporter in the six serotonergic neurons targeted by the *Sert3*-Gal4 Gal4 driver [18]. To determine whether neuronal activity in the CSD neurons is sufficient to increase attraction to 5% EtOH containing food odor blends, neuronal activity was induced by light activation using a UAS-*ChR2* (*Channelrhodopsin-2*) transgene under the control of the *RN2*-GAL4 driver (Fig 8). Control flies were fed with the vehicle whereas the experimental group with all-trans retinal. The presence of all-trans retinal transforms the ChR2 into a depolarizing blue light-gated cation selective ion channel which activates neurons [50]. In the experimental set up flies choose between a 5% EtOH containing food odor blend illuminated with blue light and source of food odor blend illuminated with yellow light (Fig 8A). As expected the control flies significantly preferred the EtOH-enriched food odor blend. Activation of the CSD neurons eliminated the attraction (Fig 8B). To address whether attraction might be induced to lower EtOH concentrations that normally do not elicit attraction, similar experiments were performed using 1% EtOH containing food odor blends (Fig 8C). Here, both groups did not show attraction. To independently address the function of neuronal activity on attraction we inhibited the activity of the CSD neurons by expression of the *UAS*-tetanus toxin (TNT) transgene under the control of the *RN2*-Gal4 driver and analyzed the attraction to 5%EtOH containing food odors (Fig 8D). The block of evoked neurotransmitter release throughout development resulted in significant loss of attraction. Thus temporary activation and blocked of evoked neurotransmitter release of the CSD neurons resulted in a similar phenotype of loss of attraction.

## Discussion

How does serotonin signaling in CSD neurons affect odorant attraction in an opposing manner, e.g., increases the attraction to a low concentration of AA and increases aversion to a higher concentration of AA? In general, the attractiveness of the key odorant AA within food odor blends is concentration dependent [4]. The activation profile of glomeruli differs depending on the concentration. Apple cider vinegar, with its main component acetic acid, activates the DM1 and VA2 glomeruli at a low concentration, and this activation is sufficient to result in approach behavior [51]. At a higher concentration of apple cider vinegar, the DM5 glomerulus is additionally activated, and this activation is sufficient to cause aversion [51]. AA might also be perceived by its acidity, and activation of the acidity-responding DP1m glomerulus causes aversion [52]. Serotonergic CSD neurons form comparable numbers of active zones with the aversion-mediating and acid-responding DP1m glomerulus and with the DM1 glomerulus that mediates attraction to a low concentration of AA [42], [52], [8], [51]. Thus, the alteration of serotonin signaling by CSD neurons might similarly activate the glomeruli independent of the concentration of AA, but the glomeruli are already in a different state of activation.

Alternatively the expression of a mutated version of the serotonin transporter during development might change the innervation pattern of the glomeruli and that in turn might alter odorant and concentration specific responses to key odorants within food odor blends.

Within the glomerulus, changes in attraction to AA are achieved by 5-HT1B receptor function. We showed that 5-HT1B acts as a positive regulator to increase the attraction to low concentrations of AA and as negative regulator to reduce the attraction to higher concentrations of AA in inhibitory GABAergic neurons that includes the LNs but not in the PNs. Therefore, GABAergic neurons might indirectly regulate the attraction by modulating the aversion- or attraction-specific glomeruli in a similar manner. The function of the 5-HT1B receptor might be shared between different odorants, since RNAi knockdown of 5-HT1B in the GABAergic neurons using the *5-HT*_*1B*_*Dro*-Gal4 driver also increases the aversion to CO_2_ and increases the attraction to female pheromones in male flies [17].

The function of the inhibitory GABAergic neurons in the regulation of attraction in flies to AA appears to be 5-HTB receptor specific, since neither knockdown of 5-HT1A nor 5-HT2A change the response to AA. This receptor specificity might be because the receptors are not expressed in the same set of inhibitory GABAergic neurons and/or have different affinities for serotonin. For example in the antennal lobes the 5-HT1A receptor is expressed in an average of 12 inhibitory GABAergic LNs, whereas the 5-HT1B receptor is expressed in an average of 4 inhibitory LNs [22]. 5-HT1A and 5-HT1B belong to the same class of serotonin receptors, but the 5-HT1B receptor has an eight-fold higher affinity for serotonin than the 5-HT1A receptor [53]. However, altered serotonin signaling in CSD neurons affects the function of5-HT1A in PN neurons but not GABAergic neurons, suggesting that the amount of serotonin secreted by CSD neurons is sufficient to alter 5-HT1A receptor function at least when higher amounts of AA are present in food odor blends. Therefore, inhibitory GABAergic are likely heterogeneous in respect to their 5-HT receptor expression.

As with the inhibitory GABAergic neurons, the PNs are also heterogeneous in respect to their 5-HT receptor function. Here, the 5-HT1A and 5-HT2A receptors but not the 5-HT1B receptor act as negative regulators for the attraction to higher AA concentrations. The function of 5-HT1A and 5-HT2A might also be dependent on the 5-HT concentration, since in response to lower AA concentrations, 5-HT1A and 5-HT2A receptor function is not required. Consistent with the idea that the PN population is heterogeneous, the GH146 Gal4 driver used to alter 5-HT receptor function targets transgene expression in at least two different kinds of PN neurons [54], [7], [55].

Within the antennal lobes, the CSD neurons form synaptic contacts with the inhibitory LNs and projection neurons [44], [42] (Fig 3). We found that CSD neurons form synapses with Kenyon cells in the calyx and indirectly via the PNs with the MBs, but olfactory short-term learning and memory and odor reversal learning are not affected by altered serotonin signaling in CSD neurons. Since the single odorants AA or EtOAc reduce the firing activity of CSD neurons [44], higher brain centers such as the LH and/or MB might provide input that activates the CSD neurons.

The concentration dependent attraction to the key odorant might be shaped by two neurotransmitters that are secreted from the CSD neurons. In addition to serotonin the CSD neurons release acetylcholine, since the CSD neurons express acetylcholine transferase and neurons with CSD morphology can be targeted by the ChAT-Gal4 driver and are serotonergic [44], (this work). Both neurotransmitter systems have opposing function on the neurons in the AL. For example the stimulation of the CSD neurons using opto-genetics results in a brief depolarization followed by a delayed hyperpolarization of the LNs [44]. The depolarization in the LNs is blocked by the nicotinic receptor antagonist, whereas the hyperpolarization by serotonin receptor antagonists [44]. Thus the release of serotonin from the CSD neurons inhibits LNs and the activation of the CSD neurons excites LNs neurons in an acetylcholinergic manner. We found that the activation of the CSD neurons using opto-genetics results in loss of attraction while blocking serotonin reuptake results in attraction to higher ethanol containing food odors that are normally less attractive. The genetic interventions differ in their timing. It might be argued that the block of serotonin uptake during development results in compensatory action and this compensation in turn results in a reduction of serotonin signaling. If this would be the case the block of serotonin release through development using TNT should phenocopy the prolonged attraction observed in flies with altered serotonin transporter function in the CSD neurons. However we found that the block of serotonin release by TNT results in loss of attraction to 5% EtOH containing food odors. These results are consistent with the idea that two neurotransmitters are secreted by the CSD neurons and regulate the attraction of the key odorant ethanol in opposing manner. Increased serotonin signaling via block of serotonin reuptake shifts the reduced or loss of attraction of higher ethanol concentrations to higher attractiveness. The release of a second neurotransmitter–probably acetylcholine- represses the attraction to 5% EtOH containing food odors. The second signal might be stronger, since opto-genetic activation of the CSD neurons results in loss of attraction. Supporting evidence that the SertDN transgene alters serotonin signaling by blocking reuptake and increasing serotonin signaling comes from the observation that loss of receptor function in GABAergic interneurons results in the opposite phenotype of altered Sert function in the CSD neurons. Here the reduction of 5-HT1B function in inhibitory GABAergic interneurons significantly reduced the attraction to a low concentration of AA (0.0125% AA; Fig 7A), whereas the expression of the SertDN transgene in the CSD neurons increases the attraction to low concentration of AA (Fig 1B). Thus the SertDN transgene is a valid tool to separate the function of the two neurotransmitters secreted by the CSD neurons.

The modulation of odorant attraction by serotonin appears to be odorant specific. Increased serotonin signaling results in higher attraction to low levels of AA level (Fig 1B) and increases attraction to higher levels of EtAc or ethanol that are normally less attractive (Fig 1C; [18]). Why is the attraction to detect and response with attraction to different key odorants regulated differentially? The smell of AA is typical for the bacteria acetobacter [3] and the small amounts of bacteria might be beneficial for flies. For example the presence of acetobacter can improve larval growth and development, when laboratory food is poor in proteins [56]. EtAc and ethanol are highly enriched odorants emitted from yeast and yeast serves as protein source for flies [2]. Higher levels of ethanol (over 10% EtOH) might be intoxicating and are aversive [6]. Under normal food condition the behavioral response needs to be balanced between the requirement for proteins and the avoidance of the aversive effect of ethanol. When flies are protein deprived they might start to approach fermenting food sources that are normally unattractive and intoxicating, since the requirements for proteins are stronger than the aversive effects of ethanol. We have previously shown that the attraction for food odor source is relative and dependence on the alternative offered [4]. Thus the internal condition of the fly might modulate the activity of the CSD neuron. In combination with the specific concentration dependent activation pattern of the different odorants in the glomeruli this might result than in a selective response to different key odorant enriched food sources.

In summary, the CSD neurons shape odorant attraction at the level of the projection neurons and GABAergic neurons. Increased serotonin signaling in the CSD neurons does not interfere with learning and memory or food consumption. Serotonin acts as a negative regulator for the attractiveness of higher concentration of AA. The regulation of the odorant attraction is achieved by different down-stream serotonin receptors expressed in different types of neurons. The 5-HT1B receptor in the inhibitory GABAergic neurons including the LNs is a positive regulator of the attraction to low concentrations of AA and a negative regulator of the attraction to higher concentrations of AA. Higher odorant concentrations also require the function of 5-HT1A and 5-HT2A receptors as negative regulators in projection neurons but not in inhibitory GABAergic neurons.

## Supporting information

S1 Fig No influence of altered serotonin signaling in subsets of central brain neurons on negative geotaxis in satiated or starved flies. **A** and **B**, The expression of the *UAS*-mCD8::SERT^DN^ transgene under the control of the *RN2*-Gal4 driver did not significantly alter negative geotaxis in satiated (**A**) or starved (**B**) flies (N = 11 different sets of flies). C and D, The expression of the *UAS*-mCD8::SERT^DN^ transgene under the control of the of *Sert3*-Gal4 driver did not significantly alter negative geotaxis in satiated (**C**)or starved (**D**) flies (N = 10–11 different sets of flies). The data are presented as the mean ± s.e.m. For the underlying numerical data see S9 Table.
(TIF)

S2 Fig The anatomical characterization of CSD neurons in the adult brain. **A**, The projections of CSD neurons are visualized with the *UAS*-mCD8::GFP transgene under the control of the RN2-Gal4 driver (in green), and the brain neuropil is labeled with the nc82 antibody serum (magenta). **B** and **C**, The postsynaptic arbors of CSD neurons are labeled with a dendritic marker DenMark (magenta) in the antennal lobe (AL), calyx and lateral horn (LH). The brain neuropil in **B** to **E** is labeled with the nc82 marker (here in green). **D** and **E**, The presynaptic arbors of CSD neurons are labeled with a presynaptic marker BR P (magenta), which is enriched in the AL (**D**), calyx and LH (**E**). Scale bars represent 50 μm.
(TIF)

S1 Table Fly stocks. (DOCX)

S2 Table Sensory acuity tests. (DOCX)

S3 Table Raw data corresponding to Fig 1. (XLSX)

S4 Table Raw data corresponding to Fig 2. (XLSX)

S5 Table Raw data corresponding to Fig 5. (XLSX)

S6 Table Raw data corresponding to Fig 6. (XLSX)

S7 Table Raw data corresponding to Fig 7. (XLSX)

S8 Table Raw data corresponding to Fig 8. (XLSX)

S9 Table Raw data corresponding to S1 Fig. (XLSX)
