# Supplementary figures and images for: Serotonin transporter dependent modulation of food-seeking behavior
Source: PLoS One. 2020 Jan 24;15(1):e0227554. doi: 10.1371/journal.pone.0227554 (PMC6980608; doi:10.1371/journal.pone.0227554)

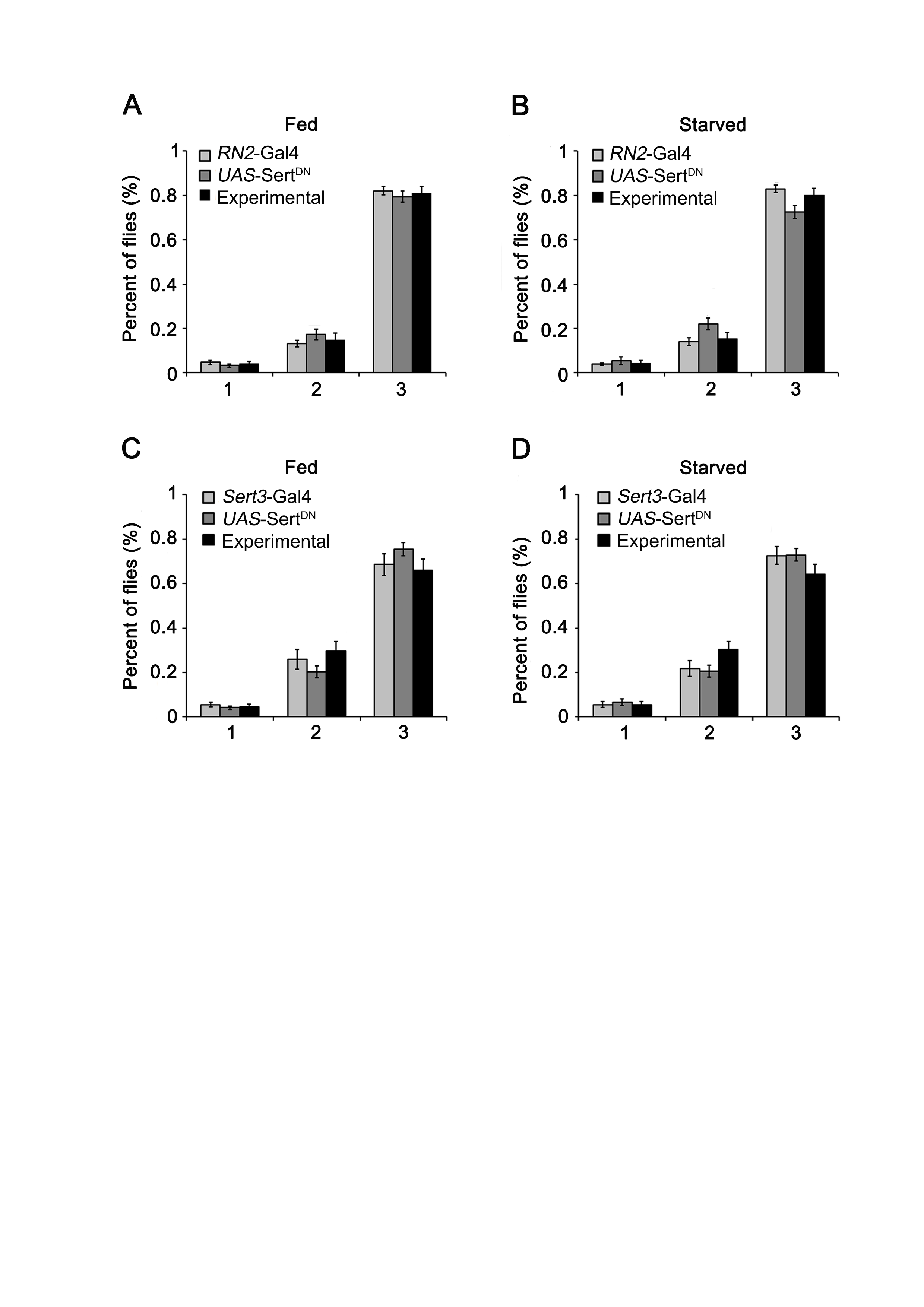

Supplement: S1 Fig — A and B, The expression of the UAS-mCD8::SERTDN transgene under the control of the RN2-Gal4 driver did not significantly alter negative geotaxis in satiated (A) or starved (B) flies (N = 11 different sets of flies). C and D, The expression of the UAS-mCD8::SERTDN transgene under the control of the of Sert3-Gal4 driver did not significantly alter negative geotaxis in satiated (C)or starved (D) flies (N = 10–11 different sets of flies). The data are presented as the mean ± s.e.m. For the underlying numerical data see S9 Table. (TIF) [file pone.0227554.s001.tif]

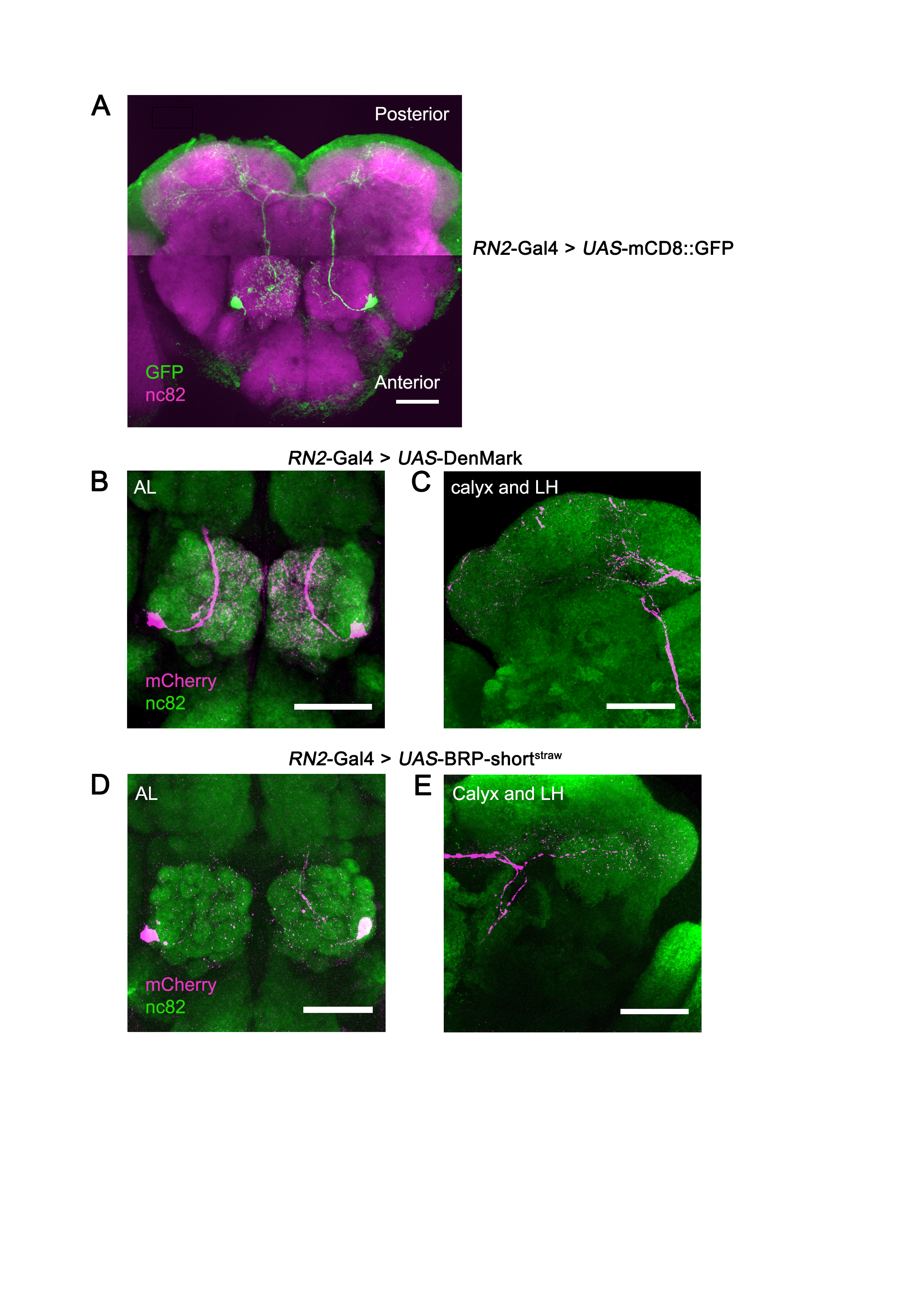

Supplement: S2 Fig — A, The projections of CSD neurons are visualized with the UAS-mCD8::GFP transgene under the control of the RN2-Gal4 driver (in green), and the brain neuropil is labeled with the nc82 antibody serum (magenta). B and C, The postsynaptic arbors of CSD neurons are labeled with a dendritic marker DenMark (magenta) in the antennal lobe (AL), calyx and lateral horn (LH). The brain neuropil in B to E is labeled with the nc82 marker (here in green). D and E, The presynaptic arbors of CSD neurons are labeled with a presynaptic marker BR P (magenta), which is enriched in the AL (D), calyx and LH (E). Scale bars represent 50 μm. (TIF) [file pone.0227554.s002.tif]
