# Supplementary material for: Serotonin transporter dependent modulation of food-seeking behavior
Source: PLoS One. 2020 Jan 24;15(1):e0227554. doi: 10.1371/journal.pone.0227554 (PMC6980608; doi:10.1371/journal.pone.0227554)
Supplement: S1 Table — (DOCX) [file pone.0227554.s003.docx]

**S1 Table: Fly Stocks**

| *Genotype* |  | *Source* |
| --- | --- | --- |
| *RN2-E-*Gal4 |  | Fujioka et al., 2003 |
| *Sert3-*Gal4 |  | Xu et al., 2016 |
| *Orco*-LexA::VP16 |  | Lai and Lee, 2006 |
| *GH146*-LexA::GAD |  | Sudhakaran et al., 2012 |
| *GMR29A12-*LexA |  | Pfeiffer et al., 2010 |
| *GAD1-*Gal4 |  | Jackson et al., 1990 |
| *mb247-*DsRed*; mb247-*CD4::spGFP11*, UAS-*CD4::spGFP1-10 |  | Pech et al., 2013 |
| *UAS*-Sert^DN^::GFP |  | Xu et al., 2016 |
| *UAS*-Sert::GFP |  | This work |
| *UAS*-*Trh*-RNAi |  | Albin et al., 2015 |
| *UAS-*BRP-short^straw^ |  | Owald et al., 2010 |
| *UAS*-mCD8::GFP |  | Lee and Luo, 2001 |
| *LexAop-*CD4::spGFP11*;*  *UAS-*CD4::spGFP1-10 |  | Gordon and Scott, 2009 |
| *LexAop-*myr::mCherry |  | Diegelmann et al., 2008 |
| *UAS-*DenMark |  | Nicolaï et al., 2010 |
| *5-HT1A*^Δ5kb^ | deletion of 3' coding region BL#27640 | [Yuan et al., 2006](http://flybase.org/reports/FBrf0195242.html) |
| *5-HT1B*^MB05181^ | Insertion in CG15113 BL#24240 | Johnson et al., 2011 |
| *5-HT2A*^MI00459^ | Insertion in CG1056 BL#31012 | Ro et al., 2016; this work |
| *5-HT7*^MB01344^ | Insertion in CG12073 BL#23066 |  |
| UAS-d5HT1A^RNAi^ | [P{KK108407}](http://flybase.org/reports/FBtp0046487.html); VDRC#106094 | Lee et al., 2011; Ries et al., 2017 |
| UAS-d5HT1B^RNAi^ | [P{UAS-5-HT1B.IR}](http://flybase.org/reports/FBtp0022542.html); BL#27634 | [Yuan et al., 2005](http://flybase.org/reports/FBrf0195242.html); Ries et al., 2017 |
| UAS-5HT2A-RNAi | 5-HT2ARNAi; BL #31882 | Gasque et al., 2013 |

**References:**

Albin SD, Kaun KR, Knapp JM, Chung P, Heberlein U, Simpson JH. 2015. A Subset of Serotonergic Neurons Evokes Hunger in Adult Drosophila. Curr Biol 25:2435-2440.

Diegelmann S, Bate M, Landgraf M. 2008. Gateway cloning vectors for the LexA-based binary expression system in Drosophila. Fly (Austin) 2:236-239.

Fujioka M, Lear BC, Landgraf M, Yusibova GL, Zhou J, Riley KM, Patel NH, Jaynes JB. 2003. Even-skipped, acting as a repressor, regulates axonal projections in Drosophila. Development 130:5385-5400.

Gasque G, Conway S, Huang J, Rao Y, Vosshall LB. 2013. Small molecule drug screening in Drosophila identifies the 5HT2A receptor as a feeding modulation target. Sci Rep 3:srep02120.

Gordon MD, Scott K. 2009. Motor control in a Drosophila taste circuit. Neuron 61:373-384.

Jackson FR, Newby LM, Kulkarni SJ. 1990. Drosophila GABAergic systems: sequence and expression of glutamic acid decarboxylase. J Neurochem 54:1068-1078.

Johnson O, Becnel J, Nichols CD. 2011. Serotonin receptor activity is necessary for olfactory learning and memory in Drosophila melanogaster. Neuroscience 192:372-381.

Lai SL, Lee T. 2006. Genetic mosaic with dual binary transcriptional systems in Drosophila. Nat Neurosci 9:703-709.

Nicolai LJ, Ramaekers A, Raemaekers T, Drozdzecki A, Mauss AS, Yan J, Landgraf M, Annaert W, Hassan BA. 2010. Genetically encoded dendritic marker sheds light on neuronal connectivity in Drosophila. Proc Natl Acad Sci U S A 107:20553-20558.

Owald D, Fouquet W, Schmidt M, Wichmann C, Mertel S, Depner H, Christiansen F, Zube C, Quentin C, Korner J, Urlaub H, Mechtler K, Sigrist SJ. 2010. A Syd-1 homologue regulates pre- and postsynaptic maturation in Drosophila. J Cell Biol 188:565-579.

Pech U, Revelo NH, Seitz KJ, Rizzoli SO, Fiala A. 2015. Optical dissection of experience-dependent pre- and postsynaptic plasticity in the Drosophila brain. Cell Rep 10:2083-2095.

Pfeiffer BD, Ngo TT, Hibbard KL, Murphy C, Jenett A, Truman JW, Rubin GM. 2010. Refinement of tools for targeted gene expression in Drosophila. Genetics 186:735-755.

Ries AS, Hermanns T, Poeck B, Strauss R. 2017. Serotonin modulates a depression-like state in Drosophila responsive to lithium treatment. Nat Commun 8:15738.

Ro J, Pak G, Malec PA, Lyu Y, Allison DB, Kennedy RT, Pletcher SD. 2016. Serotonin signaling mediates protein valuation and aging. 5.

Sudhakaran IP, Holohan EE, Osman S, Rodrigues V, Vijayraghavan K, Ramaswami M. 2012. Plasticity of recurrent inhibition in the Drosophila antennal lobe. J Neurosci 32:7225-7231.

Xu L, He J, Kaiser A, Graber N, Schlager L, Ritze Y, Scholz H. 2016. A Single Pair of Serotonergic Neurons Counteracts Serotonergic Inhibition of Ethanol Attraction in Drosophila. PLoS One 11:e0167518.

Yuan Q, Joiner WJ, Sehgal A. 2006. A sleep-promoting role for the Drosophila serotonin receptor 1A. Curr Biol 16:1051-1062.

Yuan Q, Lin F, Zheng X, Sehgal A. 2005. Serotonin modulates circadian entrainment in Drosophila. Neuron 47:115-127.
