# Supplementary material for: Serotonin transporter dependent modulation of food-seeking behavior
Source: PLoS One. 2020 Jan 24;15(1):e0227554. doi: 10.1371/journal.pone.0227554 (PMC6980608; doi:10.1371/journal.pone.0227554)
Supplement: S2 Table — (DOCX) [file pone.0227554.s004.docx]

**S2 Table. Sensory acuity tests**

|  |  |  | Odor Avoidance | | | |  |
| --- | --- | --- | --- | --- | --- | --- | --- |
|  |  |  | 3-OCT(1:80) | | MCH (1:100) | |  |
| Genotype | Sucorse Reactivity | Shock  Reactivity | Satiated | Starved | Satiated | Starved | Odor  Balance |
| *RN2*-GAL4 | 0.73±0.08a | -0.86±0.04a | -0.25±0.03a | -0.34±0.05a | -0.23±0.05a | -0.33±0.05a | 0.01±0.08 |
| *UAS*-Sert^DN^ | 0.66±0.07a | -0.85±0.03a | -0.31±0.06a | -0.37±0.05a | -0.25±0.06a | -0.32±0.05a | 0.04±0.09 |
| Experimental | 0.61±0.07a | -0.79±0.04a | -0.40±0.05a | -0.35±0.06a | -0.25±0.06a | -0.33±0.04a | 0.06±0.08 |

Mean ± s.e.m is shown. No significant differences between control and experimental groups are detected using ANOVA Turkey- Cramer post hoc analysis (*P* < 0.05). The letter “**a”** indicates significant difference from random choice as determined by One-sample sign test (*P* < 0.05). N= 8 - 12 for each test except sucrose sensitivity (N = 20).
